# Effect of high fat diet on phenotype, brain transcriptome and lipidome in Alzheimer’s model mice

**DOI:** 10.1038/s41598-017-04412-2

**Published:** 2017-06-27

**Authors:** Kyong Nyon Nam, Anais Mounier, Cody M. Wolfe, Nicholas F. Fitz, Alexis Y. Carter, Emilie L. Castranio, Hafsa I. Kamboh, Valerie L. Reeves, Jianing Wang, Xianlin Han, Jonathan Schug, Iliya Lefterov, Radosveta Koldamova

**Affiliations:** 1 Department of Environmental and Occupational Health, University of Pittsburgh, Pittsburgh, 15219 PA USA; 2 Sanford Burnham Prebys Medical Discovery Institute, Orlando, 32827 FL USA; 3 Department of Genetics, Perelman School of Medicine, University of Pennsylvania, Philadelphia, PA 19104 USA

## Abstract

We examined the effect of chronic high fat diet (HFD) on amyloid deposition and cognition of 12-months old APP23 mice, and correlated the phenotype to brain transcriptome and lipidome. HFD significantly increased amyloid plaques and worsened cognitive performance compared to mice on normal diet (ND). RNA-seq results revealed that in HFD mice there was an increased expression of genes related to immune response, such as *Trem2* and *Tyrobp*. We found a significant increase of TREM2 immunoreactivity in the cortex in response to HFD, most pronounced in female mice that correlated to the amyloid pathology. Down-regulated by HFD were genes related to neuron projections and synaptic transmission in agreement to the significantly deteriorated neurite morphology and cognition in these mice. To examine the effect of the diet on the brain lipidome, we performed Shotgun Lipidomics. While there was no difference in the total amounts of phospholipids of each class, we revealed that the levels of 24 lipid sub-species in the brain were significantly modulated by HFD. Network visualization of correlated lipids demonstrated overall imbalance with most prominent effect on cardiolipin molecular sub-species. This integrative approach demonstrates that HFD elicits a complex response at molecular, cellular and system levels in the CNS.

## Introduction

Alzheimer’s disease (AD) is a senile dementia characterized by senile plaques made of amyloid β and neurofibrillary tangles. Late Onset AD (LOAD) is considered a complex multifactorial disease^1^. The genome-wide association studies (GWAS) have identified the inheritance of *APOEε4* allele of Apolipoprotein E (*APOE*) as the major genetic risk factor for LOAD. In addition, there are at least 20 gene variants reported to modify the risk for LOAD^1–3^. Most prominent among those newly identified genetic factors is a rare variant of the gene encoding Triggering Receptor Expressed on Myeloid cells 2 (*TREM2*), which is associated with a significant increase in the risk of LOAD at a level close to the risk conferred by *APOEε4* allele^1, 4^.

Environmental and life-style factors such as stress^5^, diet^6^ and physical activity were shown to affect cognition^7^ and could also affect the risk of LOAD. Evidence suggests that high fat/high cholesterol diet (HFD) could modify the risk for LOAD. Experimental studies employing mouse models of AD revealed that HFD affects amyloid pathology and cognitive performance in adult mice^8–11^ and prenatally exposed offspring^12^. HFD also profoundly affects lipid metabolism and lipid composition in the brain^13, 14^. Thus, it has been suggested that altered lipid metabolism is closely connected to the development and progression of Alzheimer’s disease pathology^15, 16^. Recent studies examining the effect of HFD on phenotype, have demonstrated significant effect on both, inflammation and cognition^17, 18^ and the expression level of TREM2^18^. Epidemiological studies have pointed to the significant overlap between cardiovascular and LOAD risk factors, such as obesity, hypertension and type 2 diabetes, likely increasing the burden of dementia^19^ especially in midlife^20^.

Previously, we have reported that HFD significantly increased amyloid plaques and aggravated cognitive decline in APP23 transgenic mice fed HFD and the treatment with Liver X Receptor agonist ameliorated this effects^8^. Here, using the same AD mouse model we aimed to integrate the effects of HFD on the AD-like phenotype, brain transcriptome and lipidome. We report that HFD significantly worsens cognitive performance in correlation with a decreased expression of genes related to neuronal projections and synaptic transmission. Conversely, HFD increased immune transcriptome in brain and TREM2 immunoreactivity. We identified specific changes in brain lipidome associated with HFD in APP expressing mice by lipidomics assays, suggesting a role for lipid sub-species in AD-like phenotype in mice.

## Results

### High fat diet aggravates AD-like phenotype of middle-aged APP23 mice

#### Effect on memory

**Figure 1:**
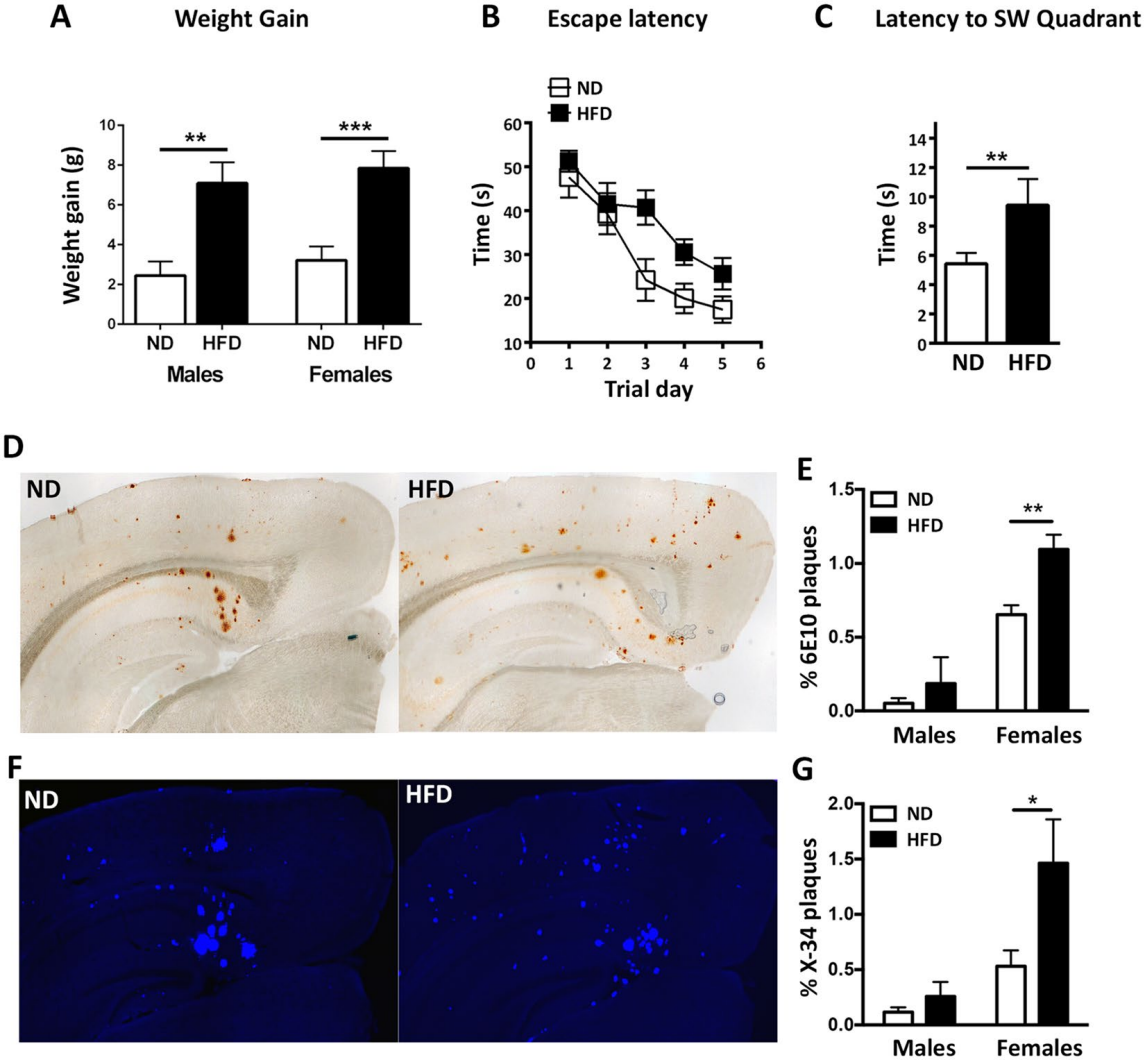
High fat diet worsens cognitive performance and increases amyloid deposition in APP23 mice. APP23 mice were fed with HFD for 3 months. (**A**) Weight gain in male and female mice. Student’s *t*-test. N = 9–11 males, 13–14 females per group. **p < 0.01; ***p < 0.001. (**B**) Acquisition trial of MWM (Escape latency). Statistic by two-way ANOVA. No interaction between trial day and diet; significant main effect of diet (F_(1,110)_ = 11.24, p = 0.0011) and trial day (F_(4,110)_ = 12.38, p < 0.0001). (**C**) Probe trial of MWM (latency to the target). Student’s *t*-test. (**B**,**C**) N = 5–7 mice gender/group. (**D–G**) Amyloid plaques in cortex and hippocampus of APP23 mice on ND and HFD were analyzed by two-way ANOVA followed by Sidak’s multiple comparison test. Representative images (**D**) and quantification (**E**) of Aβ deposits (6E10 staining). No interaction between diet and gender and significant effect of diet (F_(1,12)_ = 29.92, p = 0.0001) and gender (F_(1,12)_ = 40.58, p = 0.0001). On the graph is shown the result of Sidak’s post-hoc test. Representative images (**F**) and quantitation (**G**) of X-34 fibrillary amyloid plaques. No interaction and significant main effect of diet (F_(1,12)_ = 5.32, p = 0.004) and gender (F_(1,12)_ = 12.11, p = 0.003). On the graph is shown the result of Sidak’s post-hoc test. For D-G N = 4–5 male, 5 female mice per group. *p < 0.05 and **p < 0.01.

The goal was to test the effect of chronic feeding (HFD) on the phenotype of middle-aged AD model mice. We used one-year old APP23 mice fed HFD for three months and compared their learning and memory performance to those of mice fed normal diet (ND). We used HFD that supplies 40% of the calories from fat (milk fat and corn oil) compared to 16% fat calories in the ND. We specifically chose to start diet treatment in middle age because in one-year old APP23 mice the amyloid phenotype is present but not excessive, and thus this model is more relevant to human AD pathology at relatively early stages of the disease. As shown on Fig. 1A, weight gain resulting from HFD was similar in male and female mice. To examine cognitive performance, we used Morris Water Maze (MWM). There was no significant difference in cognitive performance between male and female mice on either diet (Supplementary Fig. S1) therefore the results are combined for both genders. As seen from Fig. 1B, HFD significantly affected the acquisition of spatial memory exemplified by the increased escape latency. Memory retention was also significantly reduced as indicated by the latency to enter the target quadrant in the probe trial of MWM (Fig. 1C).

#### Effect on amyloid deposition

Our previous data demonstrated that HFD increases amyloid deposition in APP23 mice^8^. To confirm this, total Aβ plaques (including diffuse and fibrillar) were visualized using 6E10 anti-Aβ antibody (Fig. 1D) and compact amyloid plaques assessed by X-34 staining (Fig. 1F). As visible on Fig. 1E and G, there was a significant diet and gender effect on 6E10-positive Aβ plaques as well as on X-34 labeling compact amyloid plaques, respectively. While HFD affected males and females similarly the females had significantly more amyloid in each diet group. The effect of diet on plaques was not a result of an increase in full length Aβ precursor protein (APP), as it was unaffected by HFD (Supplementary Fig. S4A and B). We also observed a significant decrease in ABCA1 protein level following HFD whereas APOE protein level was unchanged (Supplementary Fig. S4A and B). Collectively these results confirm our previous study^8^ and we conclude that in this model HFD aggravates AD-like phenotype.

### HFD affects the expression of genes involved in immune response, and those related to neuron differentiation and transcription

**Figure 2:**
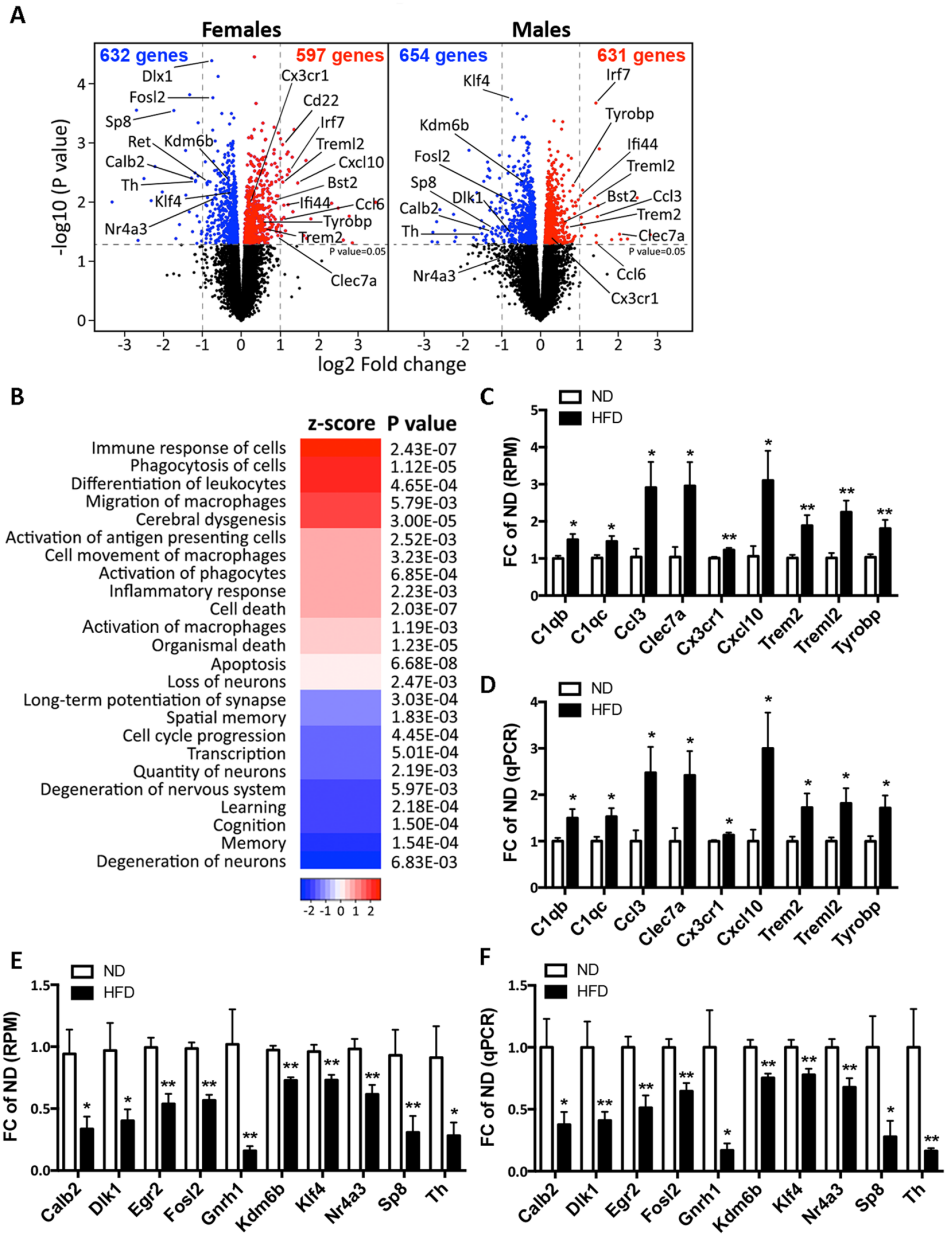
HFD affects the expression of genes associated with immune response and neuron differentiation. RNA-seq was performed on RNA isolated from cortices of APP23 mice shown on Fig. 1. ND, N = 4–6/gender; HFD, N = 5/gender. (**A**) Volcano plot representing the RNA-seq results. Red indicates up-regulated genes by HFD; blue down-regulated genes at a cutoff p < 0.05. (**B**) Heatmap representing the z-score of diseases/function annotations significantly affected by HFD (calculations by IPA). Positive z-score (red) indicates a predicted up-regulated function and negative z-score (blue) is a down-regulated function. (**C** and **D**) Significantly changed genes associated with “Immune response” and “Inflammation” in RNA-seq datasets (**C**) and qPCR validation (**D**). (**E** and **F**) Genes related to “Neuron Differentiation” and “Regulation of transcription” in RNA-seq (**E**) and qPCR validation assays (**F**). Fold change (FC) of ND was calculated from the number of reads per million (RPM) for each gene and each sample. For C and E, statistics is by edgeR. For D and F, statistics is by *t*-test, *p < 0.05; **p < 0.01. N = 9–10.

To examine if HFD affects transcriptome, we performed RNA-seq using RNA extracted from cortices of the same APP23 mice. As seen on Fig. 2A, we found similar numbers of differentially expressed genes: in females - 597 up- (red) and 632 down- (blue) regulated genes were affected by HFD, and in males - 631 up- and 654 down-regulated genes using a p-value of p < 0.05 as a cut off. Importantly, HFD induced similar changes in Gene Ontology (GO) categories in both genders (Supplementary Fig. S2A and B). GO analysis revealed that Biological Process categories most significantly up-regulated by the diet in both genders are “Immune system process” and “Innate immune response”. Most significant down-regulated category in both genders was “Transcription” and “Neuron projections” in males, and “Nervous system development” and “Neuron projection development” in females (Supplementary Fig. S2A and B).

Next, to identify commonly affected genes in males and females, we compared the lists of differentially affected transcripts in both genders. We found 301 common genes which were significantly up-regulated and 165 that were significantly down-regulated by HFD in males and females. To examine the predicted activation state of diseases and function categories, we used Ingenuity Pathway Analysis (IPA) software and calculated “z-score”. The results shown on Fig. 2B demonstrate that functions related to immune response, phagocytosis, migration of macrophages, apoptosis and inflammation had an increased activation state in the HFD group. In contrast, memory, transcription, cell cycle progression and quantity of neurons were among the most significant down-regulated functions in the HFD group.

### Validation of RNA-seq results

For further validation, we chose genes from categories commonly affected in both genders namely “Immune response”, “Neuron differentiation” and “Regulation of transcription”. The genes were assessed by qPCR, Western Blot (WB), and immunohistochemistry (IHC). Because the fold change in males and females was similar, we combined the results for both genders. On Fig. 2C and D are shown RNA-seq and qPCR results, respectively, for genes related to immune response, including *Trem2*, *Treml2*, *Tyrobp*, *Cx3cr1*, *Ccl3*, *Clec7a* and complement components. As visible from Fig. 2B, another up-regulated category in mice on HFD was apoptosis that is interconnected with immune response^21, 22^. On Supplementary Fig. S3 are shown RNA-seq results demonstrating statistically significantly increased genes by HFD, related to apoptosis - such as *Aim2*, *Sp110* and *Stat1*.

The effect of HFD on mRNA expression of genes related to neuronal differentiation and regulation of transcription is shown on Fig. 2E (RNA-seq) and F (qPCR): included are genes related to neurogenesis (*Gnrh1* and *Dlk1)*, transcription factors (*Klf4* and *Nr4a3/NOR1)*, early growth response genes (*Egr2* and *Fosl2)*, and neurotransmitter release (*Slc32a1/VGat*). *Dlk1* (delta-like homologue 1) is an atypical NOTCH ligand that is important for adult neurogenesis^23^. *Klf4* (Krüppel-like factor–4) is a transcriptional factor expressed in neural stem cells^24^ and has a role in neuronal differentiation^25^. To validate RNA-seq results we analyzed the level of proteins, coded by these genes, using WB. As seen on Supplementary Fig. S4C–F, DLK1 and KLF4 protein level was significantly decreased in mice on HFD.

We examined also protein level of vesicular GABA transporter (*Slc32a1/VGat*), which has a role in neurotransmitter release. *Slc32a1/VGat* is expressed in inhibitory GABAergic neurons and affects cognitive performance^26^. IHC results demonstrated a significant decrease of VGAT staining (Supplementary Fig. S5A–D). In contrast, HFD had no effect on mRNA and protein level of vesicular glutamate transporter *Slc17a7/VGut*, also involved in neurotransmitter release^27^ (Supplementary Fig. S5E–G). Altogether these results suggest selective HFD effect on genes regulating inhibitory and excitatory neurotransmitter release.

### HFD increases TREM2 protein level in correlation with Aβ deposition

**Figure 3:**
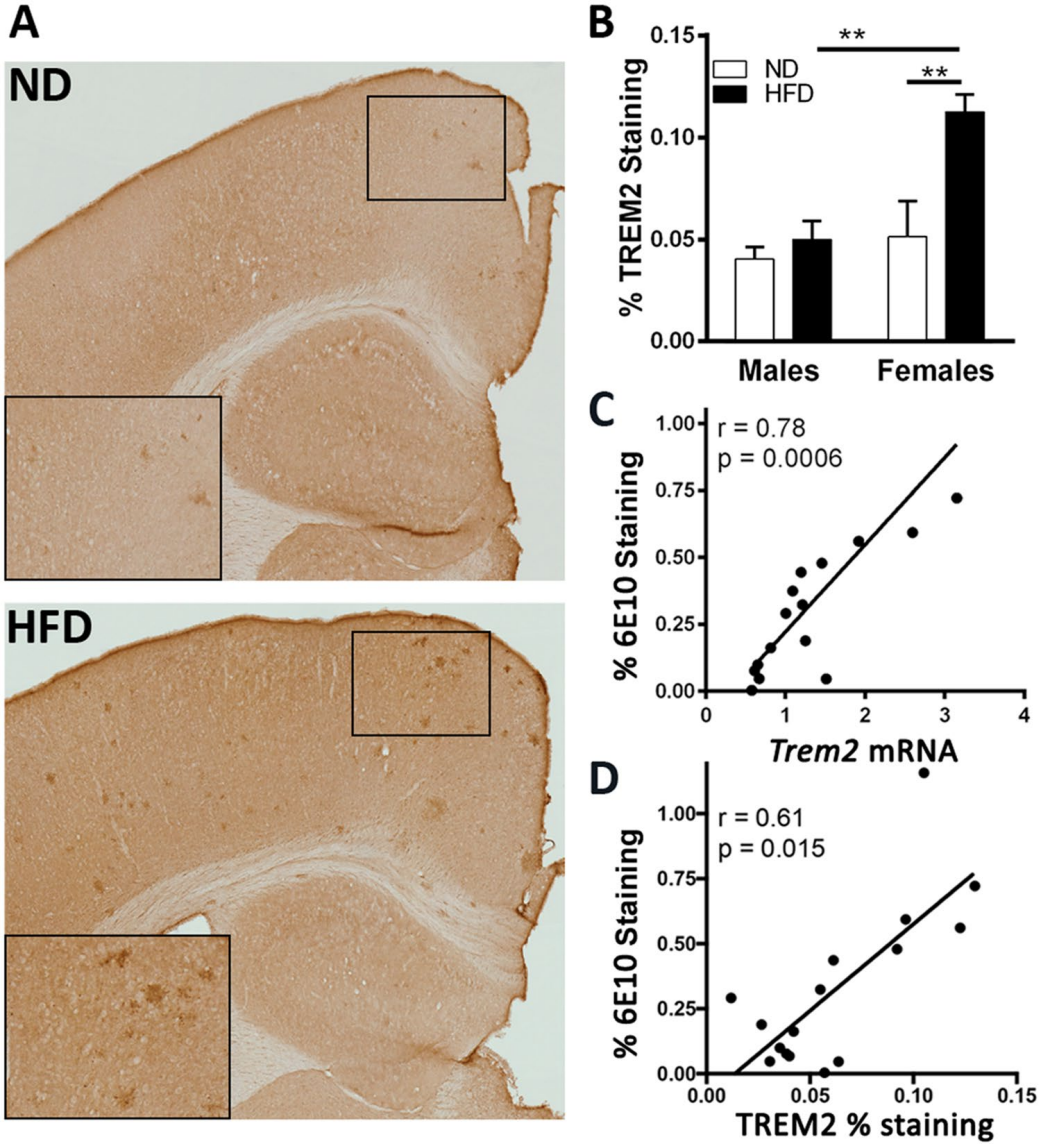
HFD increases TREM2 immunostaining in the brain. (**A**) Representative images of TREM2 staining in brains of mice fed ND or HFD. (**B**) Percentage of TREM2 stained area (N = 4 mice/gender/group). Analysis by two-way ANOVA; a significant interaction between diet and gender (F_(1,12)_ = 5.82, p = 0.0328), and a significant main effects of diet (F_(1,12)_ = 9.567, p = 0.0093) and gender (F_(1,12)_ = 11.55, p = 0.0053). **p < 0.01 by Sidak’s multiple comparison test. (**C**) Significant positive correlation between *Trem2* RNA expression and 6E10 staining (r = 0.78, p = 0.0006). (**D**) Significant positive correlation between TREM2 staining and 6E10 staining (r = 0.61, p = 0.015). Spearman’s correlation test. (N = 4/gender/group).

The importance of TREM2 as a risk factor for AD prompted further evaluation of its protein level by IHC. As shown on Fig. 3A and B, we observed a significant increase in TREM2 staining in cortex of females fed HFD compared to those on ND, and a trend toward increase in males but the difference was not significant. We also found that *Trem2* mRNA (Fig. 3C) as well as TREM2 staining (Fig. 3D) were highly correlated to the levels of 6E10-positive Aβ plaques. This data suggests that amyloid load affects *Trem2* mRNA expression and the corresponding TREM2 protein, and potentially accounts for the higher level of TREM2 in females that are more afflicted by the amyloid pathology.

### HFD affects neurite morphology

**Figure 4:**
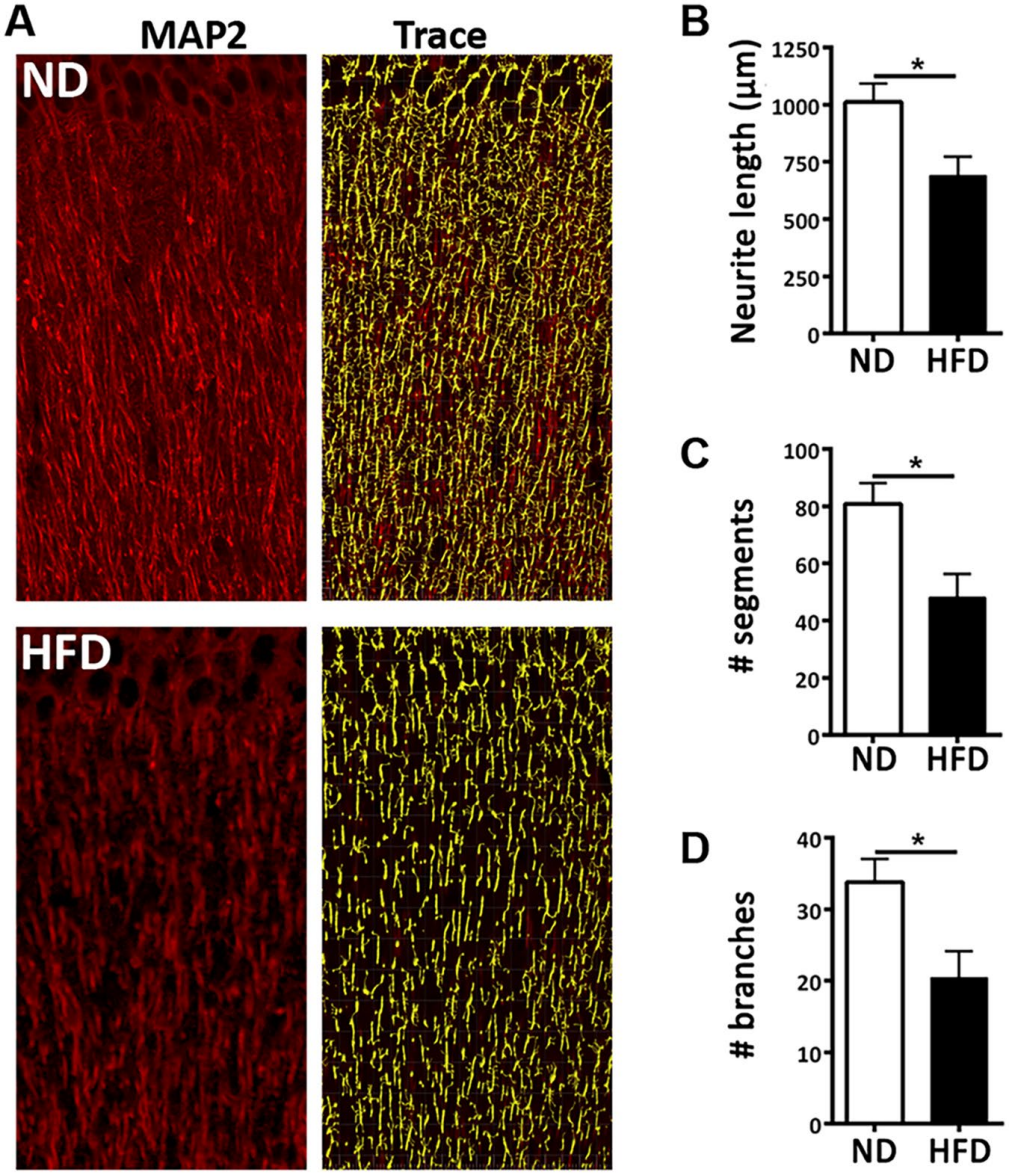
HFD impairs neurite architecture. (**A**) Representative images of MAP2 staining (red) in brain sections of ND and HFD mice with the corresponding tracing (yellow). (**B**) Average neurite length is decreased in brain of HFD compared to ND fed mice. (**C**) Average number of segments is decreased in HFD compared to ND. (**D**) Average number of branches is decreased in HFD compared to ND brains. For F, G and H, length, number of branches and number of segments are normalized on the number of nuclei (DAPI staining). N = 2 males and 3 females/group. Student’s *t*-test, *p < 0.05.

RNA-seq results revealed that HFD decreased the expression of genes related to neuron projections, axon guidance and synaptic transmission. To test if the changes in transcriptome reflect on the neuronal phenotype, we compared the dendrite architecture in ND and HFD groups using staining for neurite marker microtubule-associated protein 2 (MAP2). As shown on Fig. 4, we found that in mice fed HFD there was a significant decrease in the dendritic length (Fig. 4B) and number of segments and branches (Fig. 4C and D), suggesting that HFD detrimentally affects neurite morphology.

### HFD significantly impacts brain lipidome in AD model mice

**Figure 5:**
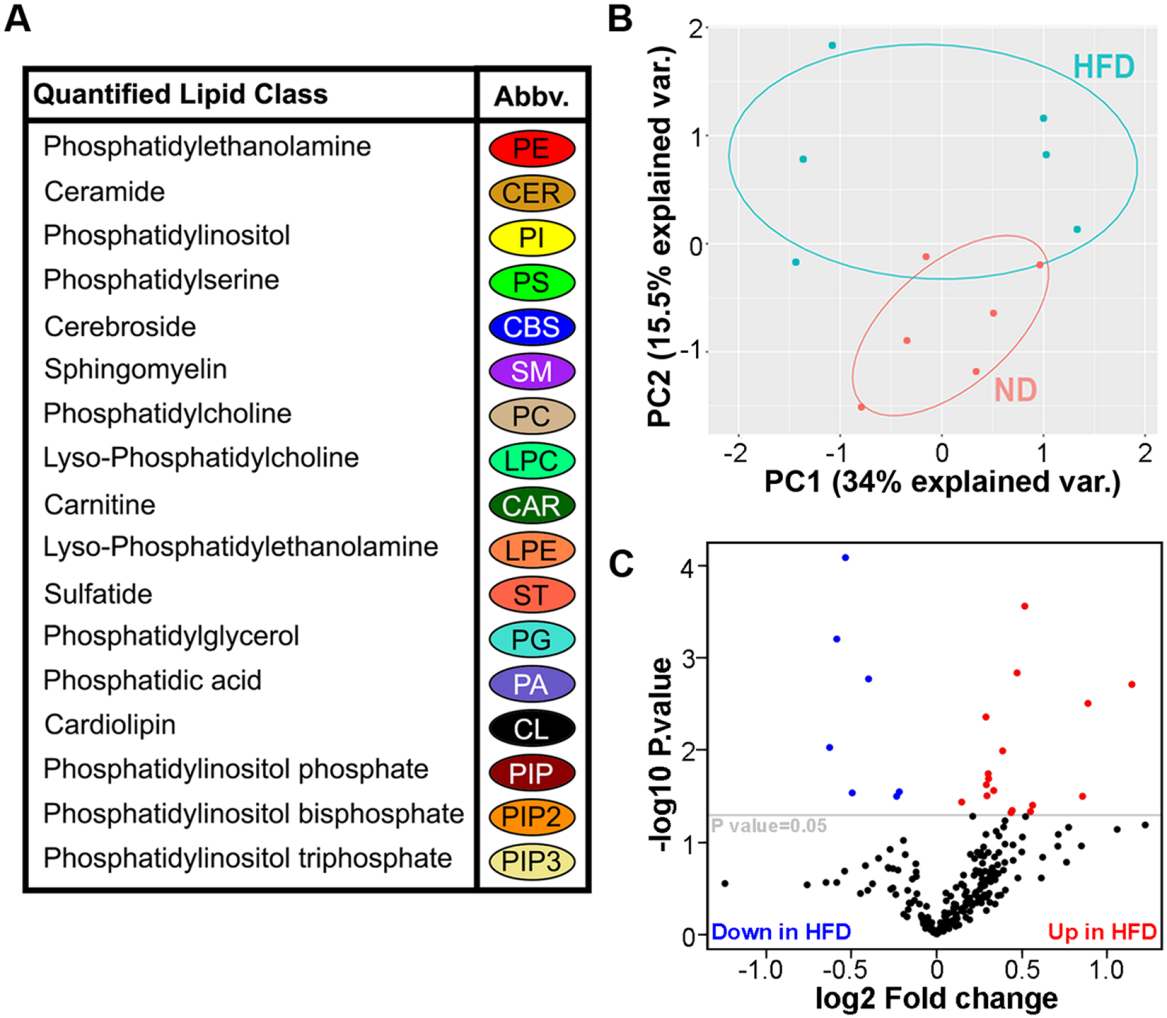
HFD significantly affects the brain lipidomic profiles. MDMS-SL was performed using cortices of APP23 mice. N = 3 male and 3 female mice per diet. (**A**) List of quantified lipid classes, their abbreviations and color association for correlation matrices. (**B**) Principal component analysis showing significant differences in brain lipidomics profiles between mice fed HFD and ND, with each point representing a single animal. (**C**) Volcano plot showing molecular species within major lipid classes significantly impacted by HFD in APP23 mice. Significant species are colored red for higher in HFD and blue for lower in HFD.

To get further insight into the effects of HFD on the phenotype, we examined brain lipidome of mice on HFD and ND using Multidimensional Mass Spectrometry based Shot Gun Lipidomics (MDMS-SL)^28^. We did not find a significant gender effect on brain lipidome and for further analysis combined the data for both genders. We identified 17 major classes of lipids (for abbreviation see Fig. 5A) comprised of 228 molecular species within those classes. We then applied Principal Component Analysis (PCA) to further process the abundance matrix of observed variables (lipid molecular species) and to calculate Principal Components that account for the highest possible variance in the datasets. We noticed that HFD samples clustered closely and were separated from ND replicates (Fig. 5B). While there was no difference in the total amounts of lipids of each class, we identified that the levels of 24 molecular species in the brain of APP23 mice were significantly modulated by HFD (Fig. 5C and Supplementary Table S1).

**Figure 6:**
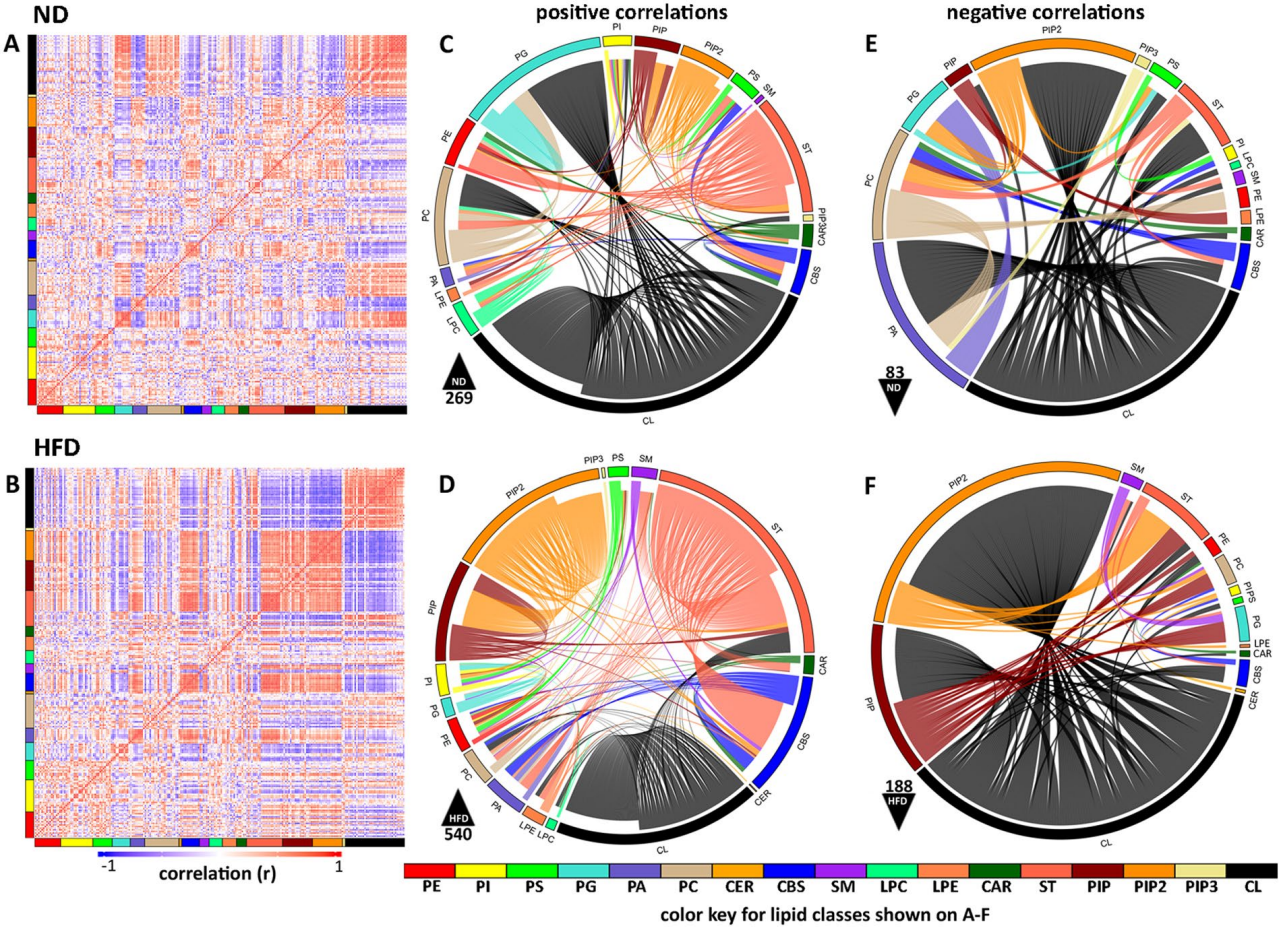
Correlation analysis of brain lipidome in HFD and ND mice. (**A**,**B**) Correlation matrix sorted by lipid class. Rows and columns correspond to 228 phospholipid species measured in ND (**A**) and HFD (**B**). Color represents the correlation level between lipid pairs. Red denotes a positive and blue a negative correlation. Each lipid class is represented by the corresponding colored bar across the bottom and left side of each square. (**C–F**) Network visualization of correlated lipid pairs in ND and HFD brains (P value > 0.005; r > 0.9 for positive and r < −0.9 for negative correlations). Chord diagrams show the positively (C and D) and negatively (E and F) correlated pairs of lipid species in ND (**C** and **E**) and HFD (**D** and **F**). Each lipid class is represented by a node around the chord diagram and color coded. Lines connecting highly correlated lipid pairs are color coded according to lipid class, with the origins of the lines of correlation being closer and termination points further from the outside nodes. In the bottom left of each diagram the triangle indicates the direction of correlation (positive or negative) and the number specifies the total number of correlations. Color key at the bottom of the figure indicated the lipid classes shown on (**A**–**F**).

To elucidate additional changes in brain lipidomes, canonical correlation analysis was performed^28^ and the correlation matrices were further processed to visualize the correlations (Fig. 6). The noticeable difference in the upper right corner of the correlation matrices (panel A–ND, panel B–HFD) indicates a change in the relationships of the related lipids (CL, ST, PIP, and PIP2). In HFD there is a stronger negative correlation of CL to ST, PIP and PIP2 than in the ND. This is accompanied by the higher positive correlation between ST, PIP, PIP2 in HFD. Network visualization of correlated lipid pairs in ND and HFD lipidomes confirmed these observations. On Fig. 6C–F the most significant correlations for both diets in the matrices (p < 0.005, r > 0.9) are presented by chord diagrams. The comparison of the negative correlations in both lipidomes clearly demonstrate that CL in HFD mice strongly and negatively correlates to PIP and PIP2 (panel F). The overall imbalance seen in HFD lipidome, when correlating the lipids in both groups is indicative of alterations by diet and is most prominent in CL, ST, PIP and PIP2 phospholipids.

### HFD significantly modulates genes associated with mitochondrial components and apoptosis

**Figure 7:**
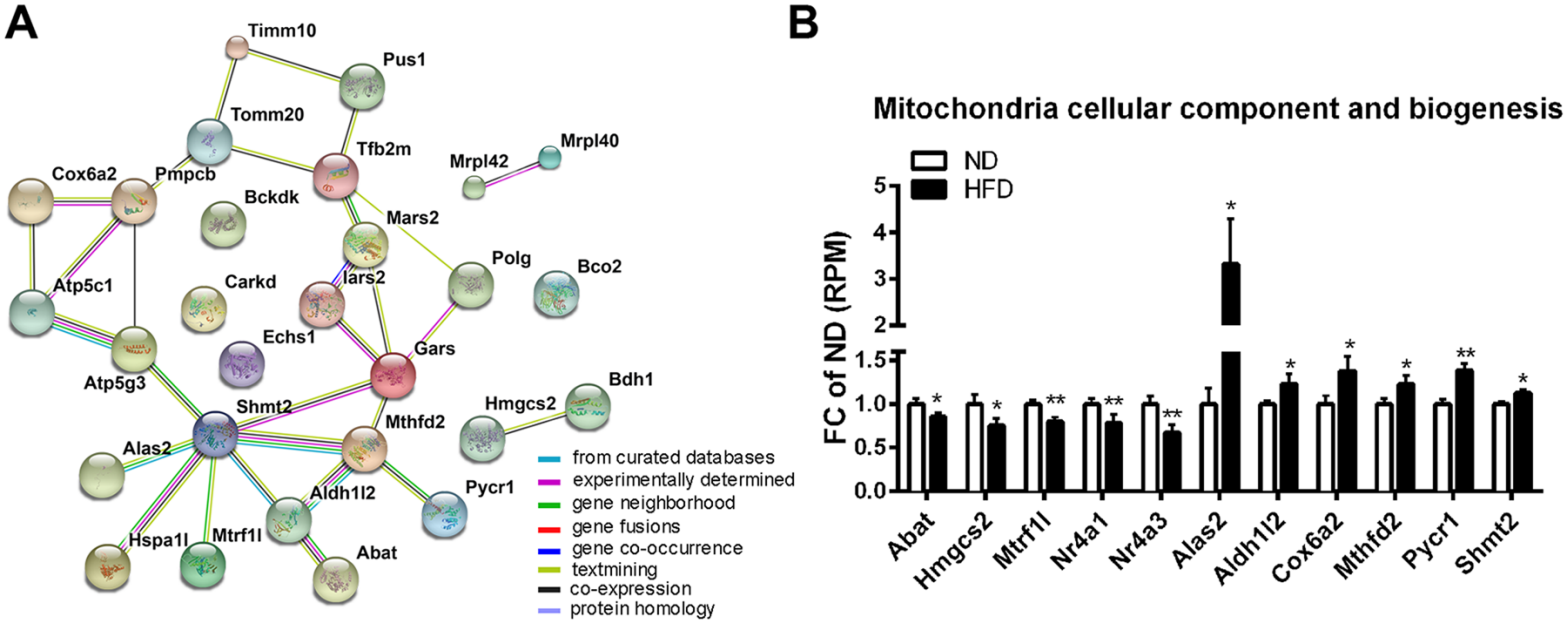
HFD significantly modulates genes associated with mitochondrial components. (**A**) Protein interaction network of significantly affected genes related to mitochondrial cellular components using String (v.10.0, http://string-db.org/). The edge represents predicted functional associations. (**B**) Representative genes of significantly changed mitochondria cellular component in RNA-seq datasets. Fold change (FC) of ND was calculated from the number of reads per million (RPM) for each gene and each sample. Statistics is by edgeR, *p < 0.05; **p < 0.01.

The phospholipid composition of cellular membranes is important for cell signaling including apoptosis and phagocytosis, and phospholipid imbalance is closely linked to mitochondrial dysfunction^29^. To have a better understanding of the interaction between transcriptome and lipidome (Figs 5 and 6), we searched our RNA-seq results for genes that code for components of mitochondrial membranes or are associated with mitochondrial biogenesis. We compared our RNA-seq data set with mitochondria cellular component genes from MSigDB (Molecular Signatures Database v5.2, http://software.broadinstitute.org/). Figure 7A shows the protein interaction network of significantly affected genes coding for proteins associated with components of mitochondrial membranes (*Cox6a2* ^30^, *Shmt2* ^31^, *Tomm20* and *Timm10* ^32, 33^) and matrix (*Abat* ^34^ and ATP synthases) as well as mitochondrial transcription factors (*Tfb2m*)^35^ by HFD. As shown the most connected protein was SHMT2 associated with mitochondrial biogenesis and immune response^31^. Among the genes related to mitochondrial component and function, most differentially affected genes by HFD were represented in Fig. 7B. Interestingly, we identified Nr4a subfamily (*Nr4a1/Nur77* and *Nr4a3/Nor1* ^36, 37^) which regulates apoptosis and inflammation was significantly decreased in HFD (Fig. 7B).

## Discussion

Our study demonstrates that HFD affects cognitive performance and amyloid pathology in APP23 transgenic mice. The diet also significantly affected the transcriptome and increased the expression level of genes related to immune response and inflammation, such as *Trem2, Tyrobp*, *P2ry12*, *Dock2*, a number of cytokines, chemokines, and *TLR*s. Conversely, the expression of the genes associated with neuronal projection development and synaptic transmission were decreased in APP23 mice fed HFD. Interestingly, in those mice there was a significant decrease of vesicular GABA transporter (*Slc32a1/VGat*), but not of *Slc17a7/VGlut*. Collectively, gene ontology categories that were significantly decreased by HFD corresponded to a phenotype presented by significantly deteriorated neurite morphology and spatial memory test. The integration of transcriptomics and lipidomics data demonstrated that the effect of HFD on behavior and AD-like phenotype in an animal model of AD is extremely multidimensional and is a result of a complex response at molecular, cellular and system levels in the CNS.

The increased mRNA level of *Trem2*, was accompanied by an increased protein level determined by IHC. Interestingly, TREM2 immunoreactivity was predominantly increased in female APP23 mice. Overall, when all mice were analyzed, there was a very high correlation between TREM2 immunostaining and Aβ plaques. The association of TREM2 with amyloid deposition is somewhat controversial and the reports, inconsistent: some groups have reported an increase and other a decrease in amyloid plaque load in a variety of animal models including *Trem2* deficient and mice expressing different TREM2 variants^38, 39^. It has been also reported that *Trem2* deficiency exacerbates amyloid pathology late in disease progression^40^, suggesting that amyloid pathology is linked to TREM2 level. A recent study demonstrated that a type of western diet enriched on carbohydrates, and different from the diet we have used, increased TREM2 immunoreactivity in association with micro- and astrogliosis^18^. Thus, an overall inflammatory response elicited by the increased amyloid deposition and diet could contribute to the worsened cognitive performance.

The main question in this study was if HFD affected gene expression/brain transcriptome and if changes in transcription profiles correlated to changes in brain lipidome? Lipids play critical roles in cellular signaling, they are major factors in membrane protein assembly, and the abnormal lipid metabolism is strongly associated with AD progression. Here, in addition to mRNA-seq we applied MDMS-SL to quantify global phospholipid changes induced by diet. The results of our lipidomics analysis demonstrate that amounts of individual molecular species of anionic phospholipid classes PS, PG and PIP3 were increased in HFD fed mice. While changes in total mass of the majority of lipid classes are important in different aspects of brain metabolism, changes in molecular speciation of PIP3 and PS are particularly important for activation of immune phagocytic receptors, phagocytosis and apoptosis^41–43^. It has been recently shown, for example, that TREM2 senses anionic lipids^38^ and TREM2 variants identified by GWAS as increasing the risk of AD, affect its binding^44^. Previous studies demonstrated that these anionic phospholipids are exposed on the outer leaflet of cell membranes and become accessible in conditions following neuronal damage inflicted by brain trauma^45^ or neurotoxic effects of Aβ^46^. In the resting cell, PS is enriched on the cytoplasmic leaflet of the plasma membrane by flippases, including P4-ATPases, and ABC transporters. However, the cells undergoing apoptosis expose PS on the cell surface and then it acts as an “eat me” signal^47, 48^. In this study we found changes in expression of several genes related to PS-mediated apoptosis–C1q (*C1qa, C1qb* and *C1qc*) (Fig. 2C and D), *Tim-3/Havcr2*, *Scarf1* and *Atp8b2* (Supplementary Fig. S3). Multiple reports by other groups suggest that C1q and TIM-3/HAVCR2 directly bind to PS on the cell surface, and C1q bound to PS provides a docking point for the scavenger receptor SCARF1. These interactions lead to increased apoptosis and initiate phagocytosis signaling pathways^49, 50^.

Perhaps the most significant changes in response to HFD, revealed in this study, were associated with CL - a major phospholipid of the inner mitochondrial membrane. Recently, it has been demonstrated that alterations of CL profile in brain of AD animal model are associated with synaptic mitochondrial dysfunction and oxidative stress^51^. In brain of HFD fed mice we identified 5 elevated CL molecular sub-species (68:4; 70;4; 72:4; 74:7; 84:20) and 2 (72:6; 76:11) with decreased amounts (Supplementary Table 1). Since the amount of CL in the outer mitochondrial membrane and intra-mitochondrial CL content are tightly regulated, significant changes in either direction from the threshold result in substantial, even detrimental consequences^52^. If these changes occur simultaneously with changes in brain transcriptome,–expression level of genes coding for proteins in the mitochondrial membranes in particular, responsible for cholesterol and protein transport^32, 33^, if there is already an undergoing neurodegenerative process, the final outcome of an external stimulus like HFD could be serious and long lasting.

Collectively, the results of our study demonstrate that HFD increases mRNA expression and protein level of genes related to immune/inflammatory response and decreases the level of genes related to neuronal differentiation and synaptic transmission. Interconnected alteration of membrane phospholipid composition and likely mitochondrial dysfunction emphasize the complexity of the neurotoxic effects of HFD, reflected in aggravated AD-like phenotype and behavioral changes that we begin to understand.

## Materials and Methods

### Animals and Diets

All animal experiments were approved by the University of Pittsburgh Institutional Animal Care and Use Committee. All procedures were carried out in accordance with the approved guidelines. APP23 transgenic mice (C57Bl6 background) expressing human APP751 familial Swedish AD mutation (APPK670N, M671L) were crossed to wild-type C57BL/6 mice to generate APP23 hemizygous mice^53^. Male and female APP23 mice (mean age 11.7 months) were randomly assigned to ND (Prolab Isopro RMH 3000, Lab Diet) or HFD (D12079B RD Western Diet, Research Diets). HFD supplemented 40% calories from fat; 16.8% from protein and 42.6% from carbohydrates. Fat was provided as anhydrous milk fat and corn oil. The mice had free access to water and food. After 3 months of feeding, behavior was assessed at mean age of 14.7 months.

### Morris Water Maze

Morris Water Maze (MWM) task was performed as previously described^8^. Briefly, the water maze pool was filled with water (20–22 °C) made opaque by non-toxic paint and visual cues were placed on the walls surrounding the pool. The hidden platform (10 cm diameter, submerged 1 cm below water level) remained in the target quadrant for the duration of testing. Performance was recorded with video tracking software (AnyMaze; Stoelting Co.). *Spatial Acquisition:* All mice performed four 60-second trials with different pool entry points and 5-minute trial intervals for five consecutive training days. *Probe Trial:* A single 60-sec probe trial was performed 24 hours after the last spatial acquisition trial where the hidden platform was removed.

### Processing of mouse brain tissue

Mice were anesthetized with Avertin (250 mg/kg of body weight, i.p.) and perfused transcardially with cold 0.1 M PBS, pH 7.4: left hemisphere fixed in 4% paraformaldehyde for histology. The cortex was dissected from the other and snap frozen on dry ice for RNA and protein isolation^54^. All procedures were as reported previously^53^. Briefly, OCT-embedded hemibrains were cut in the coronal plane at 30 μm sections and stored in a glycol-based cryoprotectant at −20 °C. Serial sections were selected 700 μm apart, starting from approximately 150 μm caudal to the first dentate gyrus appearance.

### Histology and Immunohistochemistry

All procedures were as reported previously^53^. Briefly, brain sections mounted on slides were washed in PBS and stained with X-34 (1,4-bis (3-carboxy-4-hydroxyphenylethenyl)-benzene, 100 µM, provided by W. Klunk, University of Pittsburgh). Following the staining, sections were washed in water, incubated in 0.2% NaOH in 80% unbuffered ethanol, washed again in water, and then soaked in PBS. For 6E10 staining, Adjacent sections were blocked for endogenous peroxidases, avidin-biotin quenched, antigen retrieval performed with 70% formic acid, and tissue blocked with 3% normal goat serum. Sections were then immunostained with 6E10 biotinylated antibody (1:1000, SIG-39340, Covance) at room temperature before being developed with Vectastain ABC Elite kit (Vector Laboratories, Burlingame, CA) and a DAB substrate (SK-4100, Vector Laboratories).

For neurite morphometry analysis, sectioning was performed as described above and sections sampled starting 450 μm from the first appearance of the dentate gyrus and every 360 μm (4 total sections). Sections were rinsed in PBS and permeabilized with 1% Triton X-100 in PBS for 5 hr at 4 °C. The sections were then washed in PBS and blocked with 10% goat serum at RT. The sections were incubated for 72 hr at 4 °C with MAP2 primary antibody (1:2000, MAB378, Millipore) and rinsed with PBS and then incubated with Cy3-conjugated anti-mouse secondary antibody.

TREM2 staining was performed as in refs 39 and 55. Briefly, sections were rinsed in PBS before antigen retrieval (RV1000, Biocare Medical, 1:10), endogenous peroxidases (0.3% H_2_O_2_), avidin, and biotin (PK6100, ABC Elite Vector Laboratories), and hydrophobic interactions (2% donkey serum) were blocked. Sections then incubated in TREM2 primary antibody (AF179, R&D Systems) and developed with DAB substrate. To ensure the specificity of TREM2 staining we used the brain tissue from a Trem2^KO^ mice and a staining without a primary antibody as negative control.

For VGAT and VGLUT staining, sections were incubated in either VGAT primary antibody (1:2000, AB5062P, Millipore) overnight at 4 °C or VGLUT1 primary antibody (1:1000, AB5905, Millipore) for 2 days at 4 °C before rinsing in PBS and incubating with Dylight 594 conjugated horse anti-rabbit secondary antibody (1:250; 1:1000) for 2 hr at RT. Sections were washed in PBS, mounted on slides, and counterstained with H33342 nuclear reagent.

### Quantitative Analysis

Nikon Eclipse 90i microscope (10X magnification) and Nikon NIS Element (Nikon) software were used for evaluating the amyloid pathology in the cortex and hippocampus of fixed brain sections and the pathology is presented as a percentage of area covered by X-34 or 6E10 stain. For MAP2 imaging in CA1, 4 adjacent confocal images were captured using the granule cell layer as a landmark (Olympus Fluoview 1000, 60X magnification). The FilamentTracer (Imaris, version 7.1.1, Bitplane) was utilized to determine neuronal patterning in the hippocampal CA1 region. Unbiased examination and quantitation of neurite size and length required manual introduction. Neurite length was normalized to the number of H33342 stained nuclei.

### Western blotting

Frozen cortices and hippocampi were homogenized in TBS homogenization buffer (250 mM sucrose, 20 mM Tris base, 1 mM EDTA, and 1 mM EGTA, 1 ml per 100 mg of tissue) and protease inhibitors cocktail (Roche). For WB, proteins extracted with TBS or RIPA buffer were resolved on SDS-PAGE and transferred onto nitrocellulose membranes. For APOE protein detection, TBS soluble brain extract was used and RIPA extracted proteins were used for ABCA1, APP, DLK1 and KLF4 detection. Thirty microgram of proteins were resolved on 10 or 12% SDS-PAGE gels and transferred onto nitrocellulose membranes. Used were the following primary antibodies: Anti-ABCA1 (Ab18180, Abcam), anti-ApoE (178479, Millipore), anti-APP (6E10 antibody, 9320–02, Covance), anti-DLK1 (sc-25437, Santa Cruz), anti-GAPDH (sc-25778, Santa Cruz), anti-KLF4 (sc-20691, Santa Cruz).

### RNA isolation, qPCR and sequencing

All procedures were performed as before^54, 56^. RNA was isolated from cortex and purified using RNeasy mini kit (Qiagen) per the manufacturer’s protocol. RNA quality was assessed using 2100 Bioanalyzer (Agilent Technologies) and samples with RIN > 8 were further used for sequencing. Sequencing libraries were generated using mRNA Library Prep Reagent Set (Illumina) according to the manufacturer’s recommendations. The libraries were sequenced on Illumina HiSeq2000 at the Functional Genomics Core, UPenn, Philadelphia (http://fgc.genomics.upenn.edu/). All sequencing datasets, FASTQ files in this study have been processed using Subread-featureCounts-limma/voom pipeline^57, 58^ and the differential expression analyzed by edgeR (v3.14.0) package http://bioconductor.org/packages/release/bioc/html/edgeR.html in R (v3.2.4).

For qPCR validation, cDNA was synthesized using EcoDry™ Premix, Random Hexamers (Clontech, Mountain View). qPCR was performed using TaqMan^®^ Universal Master Mix II (Life Technologies).

### Functional pathway analysis

Functional annotation clustering was performed using the Database for Annotation, Visualization and Integrated Discovery (DAVID v6.8, https://david-d.ncifcrf.gov)^59^, Ingenuity® Pathway Analysis (IPA®, QIAGEN)^60^. Gene-association network of interest was visualized using String (v10.0, http://string-db.org/).

### Multi-dimensional Mass Spectrometry-based Shotgun Lipidomics (MDMS-SL)

Quantitative analysis was performed on a triple-quadrupole mass spectrometer (Thermo Fisher Scientific) equipped with an automated nanospray apparatus NanoMate and Xcalibur system^61–63^. Internal standards for quantification of individual molecular species of the major lipid classes are added to each brain tissue sample at the start of extraction procedure. Liquid Chromatography/Electro Spray Ionization Lipidomics–Mass Spectrometry (LC/ESI-MS) Lipidomics was performed on a Dionex HPLC system/UltiMate 3000 mobile phase pump, equipped with degassing unit and autosampler^43, 64, 65^.

### Visualization of Lipidomics data

PCA and volcano plots visualized using R packages “ggbiplot2” (v2.1.0, https://github.com/vqv/ggbiplot). The correlation matrices are visualized using the package “circlize” (v0.3.8, https://github.com/jokergoo/circlize) utilizing only the significant positively (r > 0.9) and negatively (r < −0.9) correlated lipid pairs.

### Statistical analysis

The results in this study are reported as means ± SEM. To assess interaction between gender and diet, two-way repeated measures ANOVA was used. To assess interaction between gender, diet and trial day, three-way repeated measures ANOVA was used. Differences were considered significant when p < 0.05. Unless otherwise indicated all statistical analyses were performed in GraphPad Prism version 7.0. or R version 3.3.1 as indicated in the text.

## Electronic supplementary material

Supplementary information
