# Supplementary material for: Effect of high fat diet on phenotype, brain transcriptome and lipidome in Alzheimer’s model mice
Source: Sci Rep. 2017 Jun 27;7:4307. doi: 10.1038/s41598-017-04412-2 (PMC5487356; doi:10.1038/s41598-017-04412-2)

**Supplementary information**

**Effect of high fat diet on phenotype, brain transcriptome and lipidome in Alzheimer’s model mice**

Kyong Nyon Nam^1^, Anais Mounier^1^, Cody M. Wolfe^1^, Nicholas F. Fitz^1^, Alexis Y. Carter^1^, Emilie L. Castranio^1^. Hafsa I. Kamboh^1^, Valerie L. Reeves^1^, Jianing Wang^2^, Xianlin Han^2^, Jonathan Schug^3^, Iliya Lefterov^1*^ & Radosveta Koldamova^1,*^

| **Lipid subspecies (nmol/mg protein)** | **Cont (N=6)** | **HFD (N=6)** | **P value** |
| --- | --- | --- | --- |
| Phosphatidylcholine (PC) |  |  |  |
| D18:1-18:2/D16:0-20:3 | 0.6433 ± 0.0232 | 0.4433 ± 0.0212 | 0.0001 |
| D18:0-20:3 | 0.2017 ± 0.0087 | 0.1533 ± 0.0067 | 0.0017 |
| D16:0-18:2 | 1.1900 ± 0.0460 | 1.0217 ± 0.0464 | 0.0284 |
| D14:0-16:0 | 0.2517 ± 0.0128 | 0.3483 ± 0.0187 | 0.0015 |
| A16:0-18:0 | 0.3217 ± 0.0098 | 0.3567 ± 0.0099 | 0.0368 |
| Phosphatidylinositol (PI) |  |  |  |
| 16:1-20:4 | 0.1100 ± 0.0045 | 0.0717 ± 0.0065 | 0.0006 |
| 18:0-22:5 | 0.1400 ± 0.0097 | 0.1717 ± 0.0075 | 0.0239 |
| 18:2-22:6 | 0.0800 ± 0.0045 | 0.1100 ± 0.0121 | 0.0479 |
| Cardiolipin (CL) |  |  |  |
| 20:4-18:1-18:1-16:0/ 20:3-18:1-18:1-16:1/ 20:3-18:1-18:1-16:1 | 0.2050 ± 0.0205 | 0.1333 ± 0.0115 | 0.0094 |
| 20:4-20:4-18:2-18:1 | 0.2800 ± 0.0273 | 0.1967 ± 0.0167 | 0.0292 |
| 20:2-18:1-18:1-16:0/ 20:1-18:1-18:1-16:1 | 0.2017 ± 0.0119 | 0.4450 ± 0.0581 | 0.0019 |
| 18:1-18:1-16:0-16:1 | 0.0283 ± 0.0031 | 0.0467 ± 0.0056 | 0.0031 |
| 20:4-20:4-22:6-22:6 | 0.0150 ± 0.0034 | 0.0267 ± 0.0033 | 0.0318 |
| 20:4-18:1-18:1-18:1 | 0.3900 ± 0.0273 | 0.5300 ± 0.0557 | 0.0453 |
| 20:2-18:1-16:1-16:0/ 18:1-18:1-18:1-16:1 | 0.2067 ± 0.0080 | 0.3017 ± 0.0408 | 0.0466 |
| Phosphatidylethanolamine (PE) |  |  |  |
| P16:0-22:6/D18:0-18:0/P18:2-20:4 | 12.2183 ± 0.6178 | 10.3850 ± 0.3983 | 0.0319 |
| D18:1-20:4D/16:0-22:5 | 2.5533 ± 0.1463 | 3.1533 ± 0.1623 | 0.0205 |
| Phosphatidylserine (PS) |  |  |  |
| 18:1-22:6 | 1.2633 ± 0.0454 | 1.5433 ± 0.0608 | 0.0044 |
| 18:0-22:5 | 1.9033 ± 0.0952 | 2.7233 ± 0.1160 | 0.0003 |
| 20:0-20:4/18:0-22:4 | 3.0617 ± 0.1420 | 3.7767 ± 0.2102 | 0.0181 |
| Phosphatidylglycerol (PG) |  |  |  |
| 16:0-16:0 | 0.0983 ± 0.0031 | 0.1300 ± 0.0089 | 0.0102 |
| 16:0-18:1 | 0.5850 ± 0.0141 | 0.7183 ± 0.0520 | 0.0314 |
| Phosphatidylinositol tisphosphate (PIP_3_) |  |  |  |
| 18:0-20:4 | 1.3183 ± 0.0982 | 1.6617 ± 0.0910 | 0.0276 |
| Acyl Carnitine (CAR) |  |  |  |
| 16:1 | 2.6100 ± 0.3389 | 3.8533 ± 0.4031 | 0.0397 |

**Supplementary Table S1 (Related to Fig. 6). The list of 24 significantly affected lipid subspecies by HFD.** The prefixes in front of number indicate the chain linkage – A, alkyl ether; D, diacyl; P, vinyl ether plasmenyl. Amount of lipid subspecies (nmol/mg) is mean ± S.E.M. Statistics is by Student’s t-test, p<0.05.

**Supplementary Figure. 1 (Related to Fig. 1). HFD significantly affected the acquisition of spatial memory in both female and male APP23 mice.** Females, N=5-7; Males, N=5-6. Statistic is by three-way ANOVA.


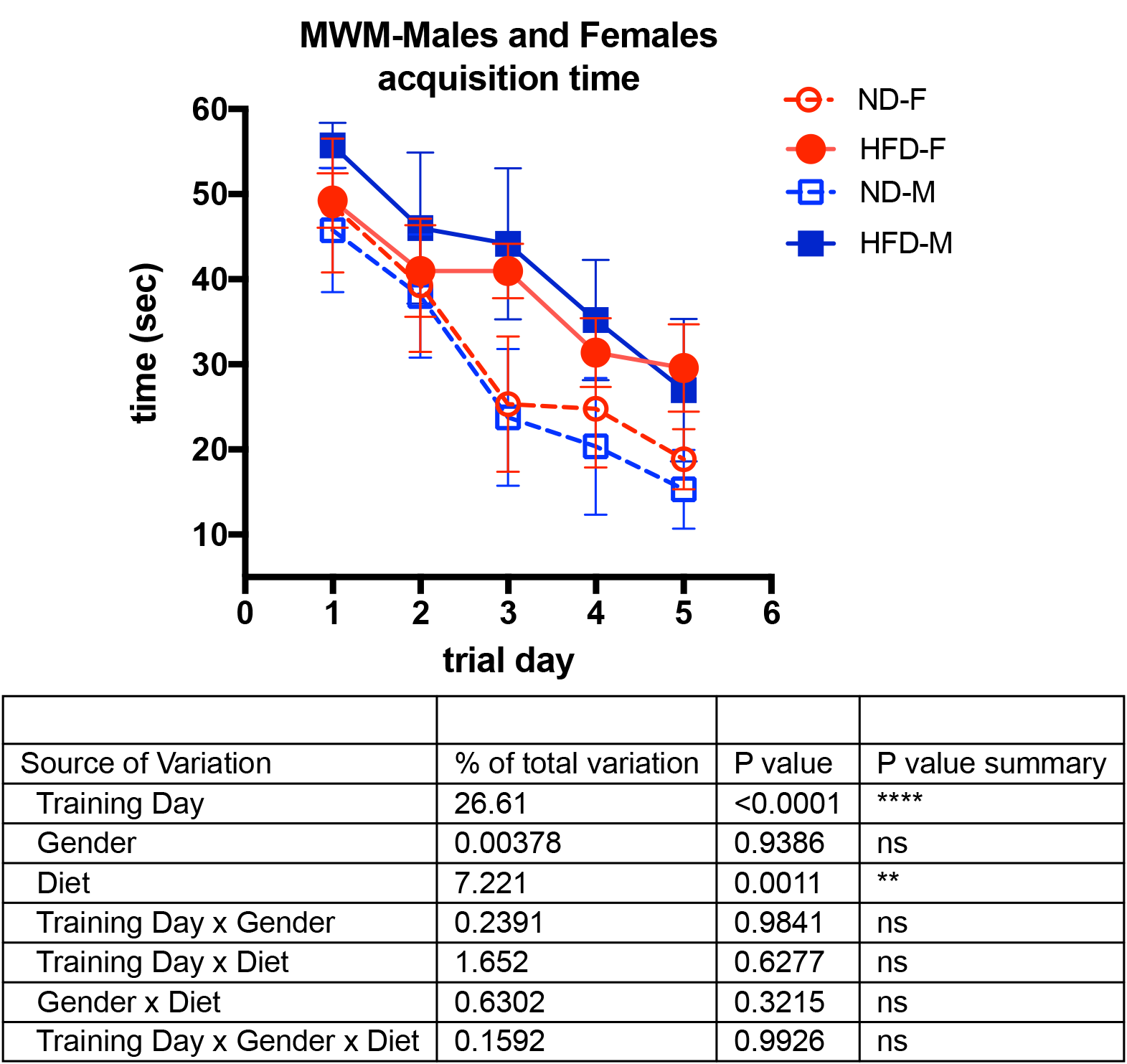


**Supplementary Figure. 2. (Related to Fig. 2). HFD alters the brain transcriptome similarly in both female and male APP23 mice.** The bubble plot shows “Biological Process” (BP) categories significantly affected by diet in female **(A)** and male **(B)** mice. The gene lists were derived from edgeR, p<0.05. Shown are significant distinct BP categories by diet as determined using DAVID. Color of bubble indicates p.value and size of bubble denotes number of significant genes in the respective category. Red label indicates commonly up-regulated categories between gender and blue label is commonly down-regulated categories.


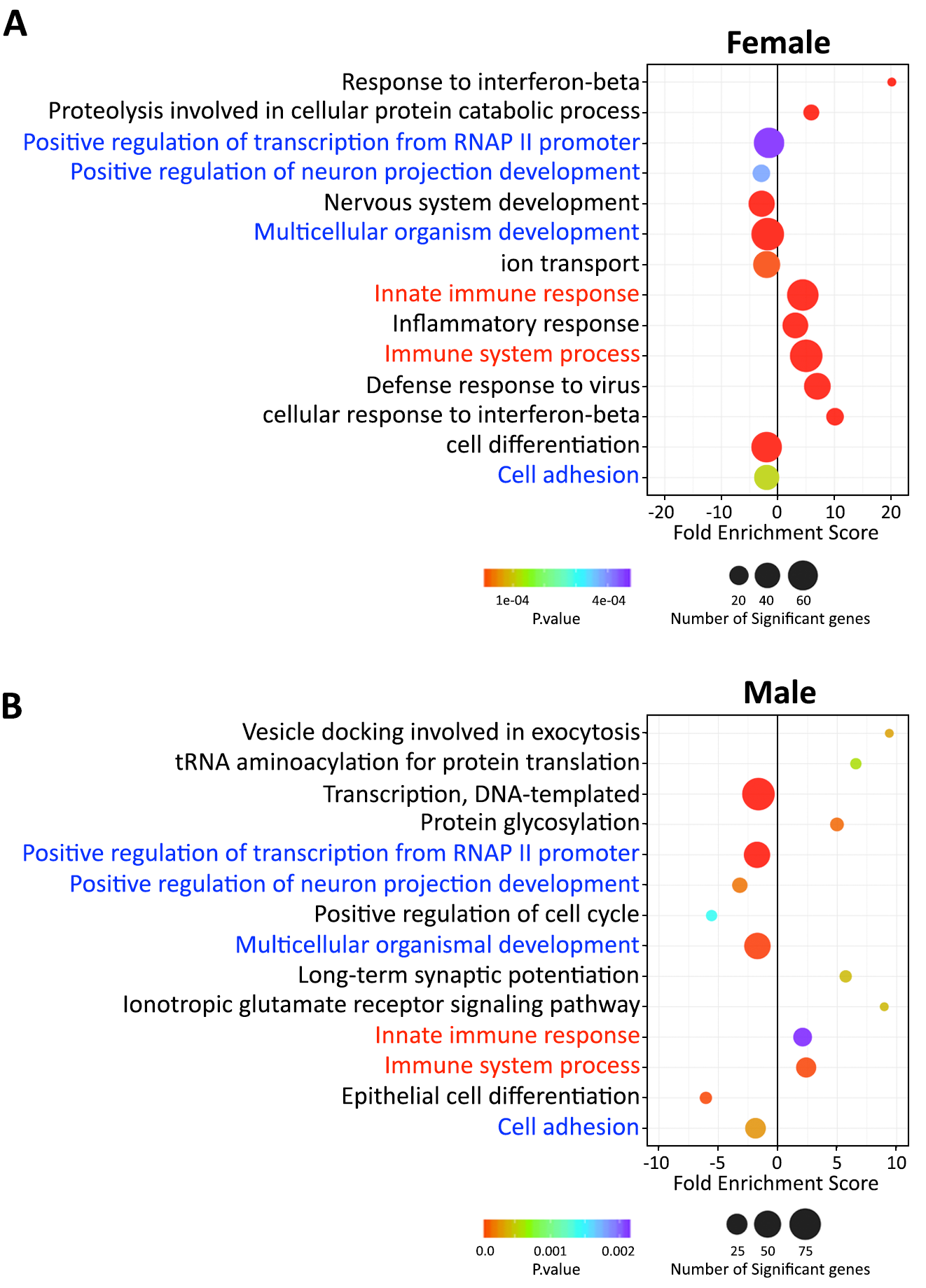


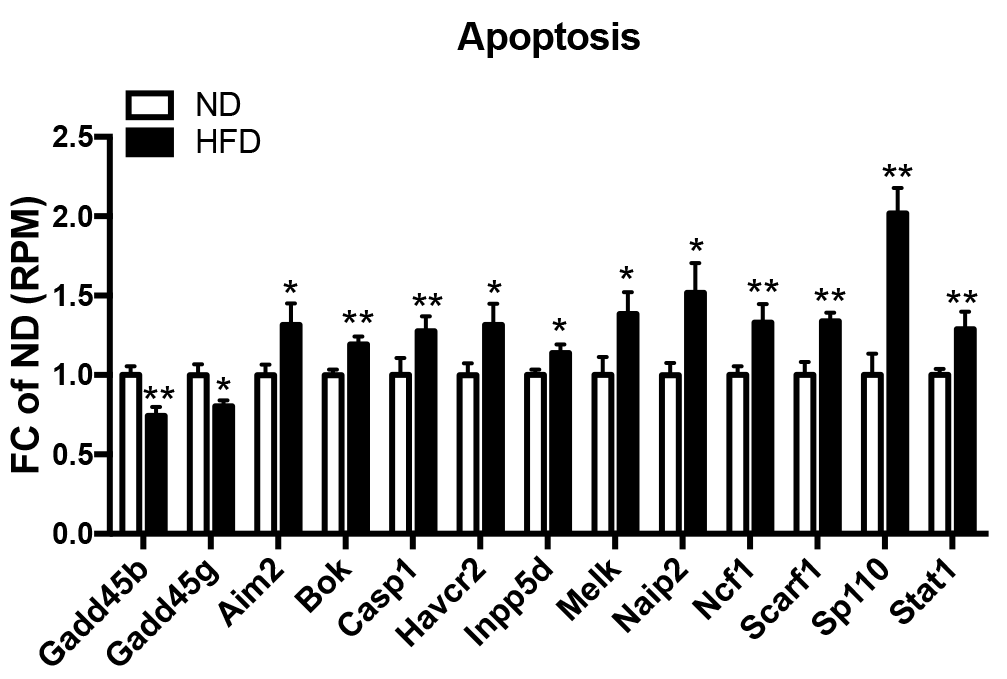


**Supplementary Figure. 3 (Related to Fig. 2). HFD significantly modulates genes associated with apoptosis.** Genes that are significantly increased by HFD were selected from diseases/function annotations by IPA (p = 4.46E-11, z-score=2.046, See Fig. 2B). Fold change (FC) of ND was calculated from the number of reads per million (RPM) for each gene and each sample. Statistics is by edgeR, *, p<0.05; **, p<0.01.


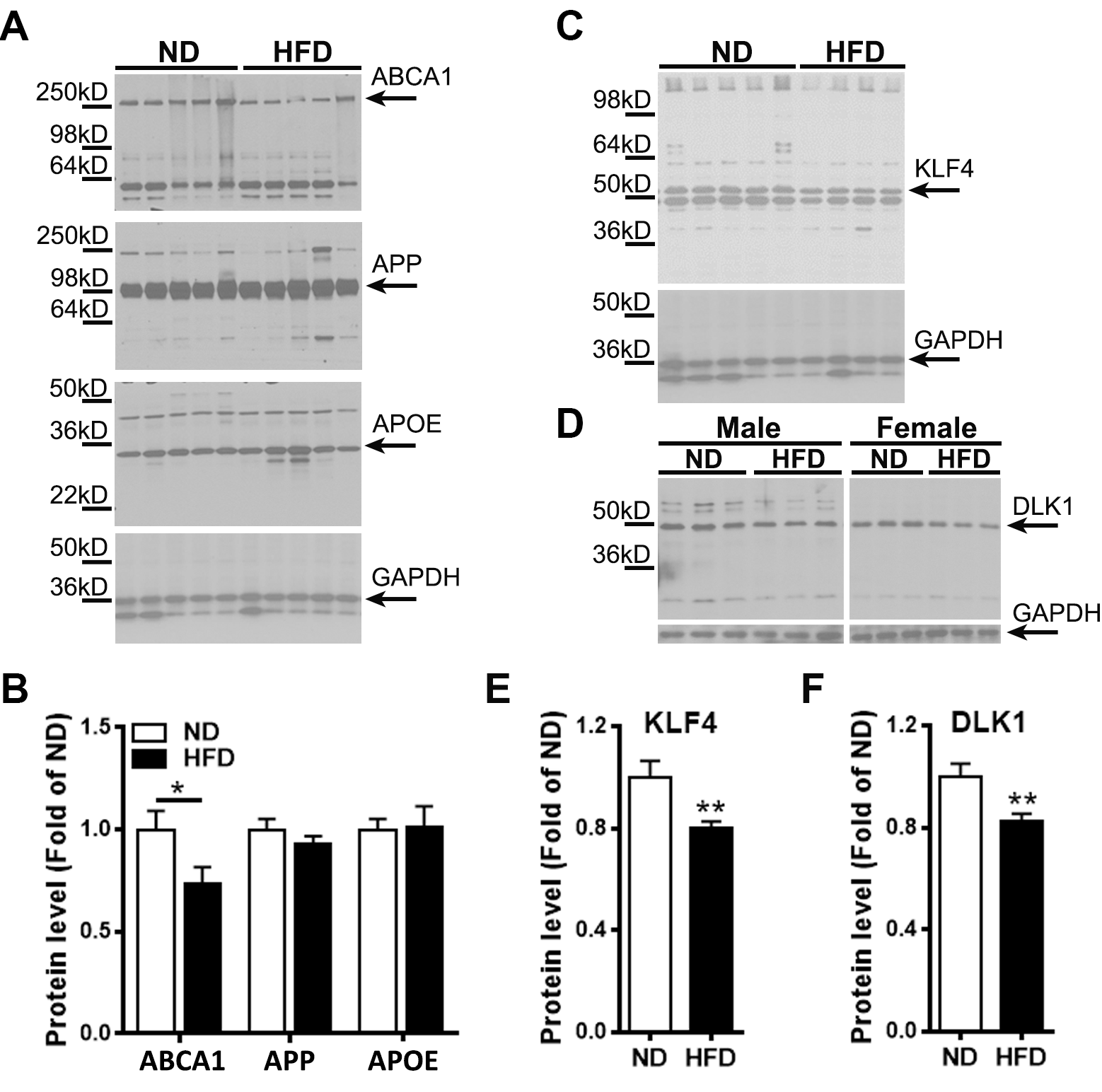


**Supplementary Figure 4 (Related to Fig. 2).** **HFD induces a decrease of protein expression of ABCA1, DLK1 and KIF4 but does not affect APP and APOE**. **(A** and **B)** HFD decreases ABCA1 protein level but does not affect APP and APOE. Representative images (A) and quantitation (B) of WB for ABCA1, APP, APOE. GAPDH is used as a loading control. Data are presented as fold of ND. N=4-5 mice/gender/group. Student’s *t*-test, *, p < 0.05. **(C** - **F)** HFD decreases DLK1 and KFL4 protein level. Representative image of DLK1 and KLF4 Western blots (C and D, respectively) and quantitation of protein levels (E and F) from male and female mice. GAPDH is used as a loading control. N=4-6 mice per gender/group. Statistics is by Student’s *t*-test, **, p < 0.01.

**Supplementary Figure. 5 (Related to Fig. 2). HFD decreases VGAT/SLC32A1 but not VGLUT/SLC17A7 protein level in the brain.** **(A-D)** HFD decreases VGAT/SLC32A1 protein and mRNA level. (A) Representative images of VGAT staining in ND and HFD mice brain. (B) Decreased percent VGAT staining in brains of mice fed HFD compared to ND. Significant decrease of VGat expression in brains of HFD fed mice. Vgat/Slc32a1 gene expression is presented as fold of ND from the RNA-seq results (C) and qPCR (D). RPM, reads per million. **(E-G)** HFD does not affect VGLUT/SLC17A7 protein and mRNA level. (E) Representative images and quantification (F) of VGLUT staining in brains of ND and HFD mice. (G) Vglut mRNA level as fold of ND from the RNA-seq results (RPM). For B and F, p value calculated by Student’ t-test, * p<0.05. N= 2 male and 3 female mice per group.


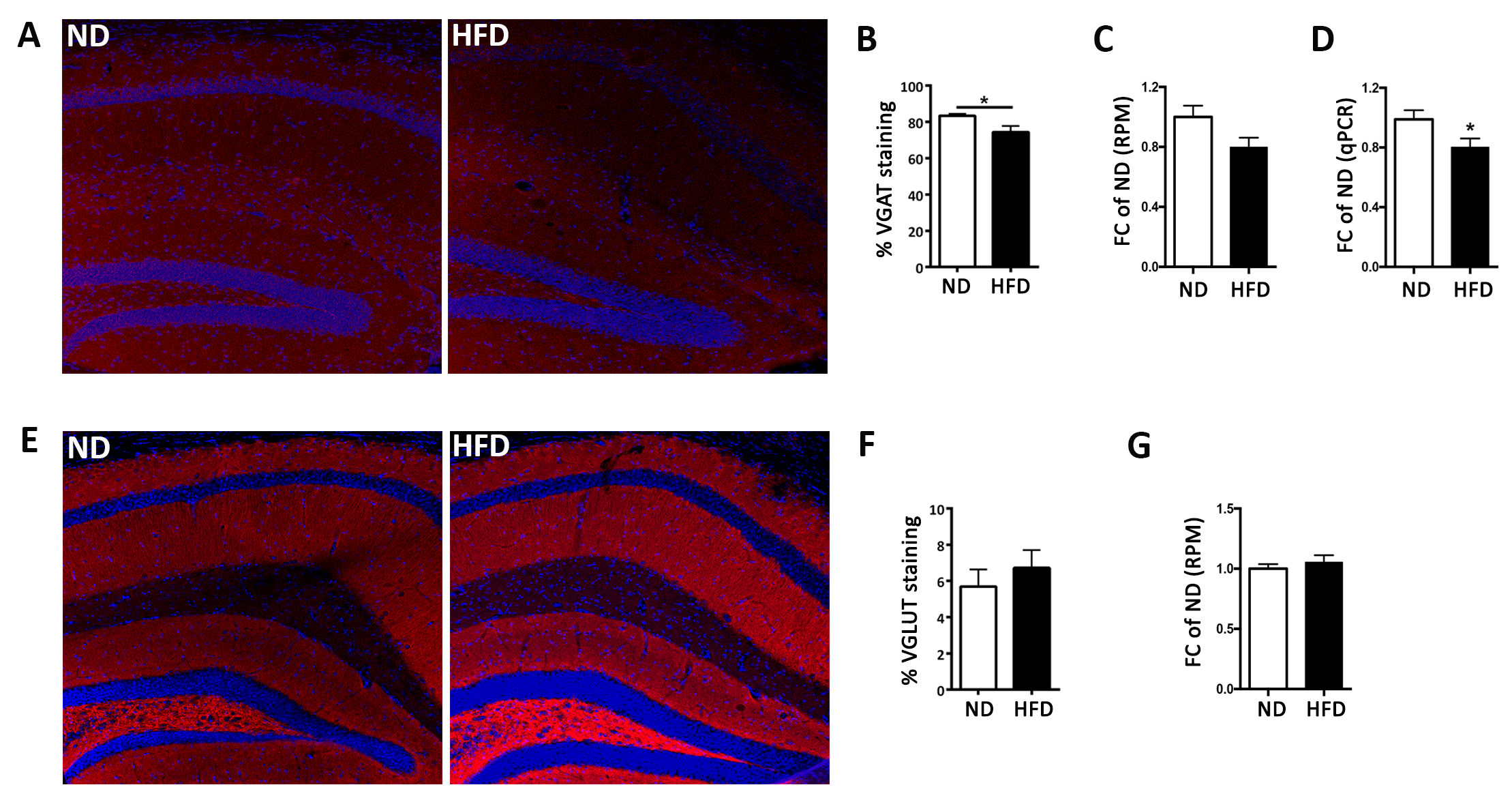

Supplement: Supplementary file 1 — Supplementary information [file 41598_2017_4412_MOESM1_ESM.doc]
